# Research Progress on Quality Detection of Livestock and Poultry Meat Based on Machine Vision, Hyperspectral and Multi-Source Information Fusion Technologies

**DOI:** 10.3390/foods13030469

**Published:** 2024-02-02

**Authors:** Zeyu Xu, Yu Han, Dianbo Zhao, Ke Li, Junguang Li, Junyi Dong, Wenbo Shi, Huijuan Zhao, Yanhong Bai

**Affiliations:** 1 College of Food and Bioengineering, Zhengzhou University of Light Industry, Zhengzhou 450000, China; happyzeyu123@126.com (Z.X.); hanyuatt@163.com (Y.H.); zhaodb212@163.com (D.Z.); like@zzuli.edu.cn (K.L.); 2014081@zzuli.edu.com (J.L.); dongjy1999@163.com (J.D.); wenbo836@163.com (W.S.); 2 Key Laboratory of Cold Chain Food Processing and Safety Control (Zhengzhou University of Light Industry), Ministry of Education, Zhengzhou 450000, China; 3 Henan Key Laboratory of Cold Chain Food Quality and Safety Control, Zhengzhou 450000, China; 4 Food Laboratory of Zhongyuan, Luohe 462000, China; 5 Henan Lianduoduo Supply Chain Management Co., Ltd., Hebi 458000, China; ldd8688@126.com

**Keywords:** livestock and poultry meat, quality detection, machine vision, hyperspectral, multi-source information fusion

## Abstract

Presently, the traditional methods employed for detecting livestock and poultry meat predominantly involve sensory evaluation conducted by humans, chemical index detection, and microbial detection. While these methods demonstrate commendable accuracy in detection, their application becomes more challenging when applied to large-scale production by enterprises. Compared with traditional detection methods, machine vision and hyperspectral technology can realize real-time online detection of large throughput because of their advantages of high efficiency, accuracy, and non-contact measurement, so they have been widely concerned by researchers. Based on this, in order to further enhance the accuracy of online quality detection for livestock and poultry meat, this article presents a comprehensive overview of methods based on machine vision, hyperspectral, and multi-sensor information fusion technologies. This review encompasses an examination of the current research status and the latest advancements in these methodologies while also deliberating on potential future development trends. The ultimate objective is to provide pertinent information and serve as a valuable research resource for the non-destructive online quality detection of livestock and poultry meat.

## 1. Introduction

Meat products are a primary source of nutrients for the human body. The protein, fat, vitamins, and other components present in livestock and poultry meat are highly similar to and easily digestible and absorbable by the human body, making them highly nutritionally valuable. In recent years, outbreaks of swine fever and avian influenza, as well as incidents involving spoiled meat, adulterated meat, added meat, and water-injected meat, have increased the emphasis on quality detection of livestock and poultry meat for both production enterprises and consumers [1]. To achieve online quality detection of livestock and poultry meat, scholars have conducted extensive research and proposed non-destructive detection methods based on machine vision technology and hyperspectral technology. Machine vision technology involves capturing the color information of livestock and poultry meat and processing these data to obtain color-based quality evaluation information. Hyperspectral technology, on the other hand, involves acquiring spectral data of livestock and poultry meat at different wavelengths and extracting relevant information based on spectral characteristics for quality evaluation. While single detection methods may achieve high success rates in experiments, their models often exhibit high specificity and poor adaptability, which limits their ability to comprehensively and accurately describe the quality characteristics of livestock and poultry meat in actual production scenarios [2]. However, the majority of current livestock and poultry meat quality detection still relies on single detection methods, leading to potential food safety issues. To address these challenges, it is necessary to comprehensively acquire and measure various aspects of sample quality information, and multi-source quality information fusion technology offers an effective solution. Multi-source quality information fusion involves utilizing multiple sensing information resources to obtain multidimensional information describing different quality characteristics of the same object. By analyzing, selecting, integrating, and balancing this information using detection algorithms, several simplified optimal composite variables can be obtained. Compared to the single detection method, multi-source quality information fusion offers advantages such as higher information content, better fault tolerance, increased accuracy, and similarity to human cognitive processes [3]. Based on this, this article provides an overview of the research progress in machine vision technology, hyperspectral technology, and multi-source quality information fusion technology for non-destructive online quality detection of livestock and poultry meat. It also discusses future development trends, aiming to provide relevant information and research references for accurate identification and non-destructive online detection of livestock and poultry meat quality.

## 2. Quality Detection of Livestock and Poultry Meat Based on Machine Vision Technology

### 2.1. Machine Vision Technology

**Figure 1:**
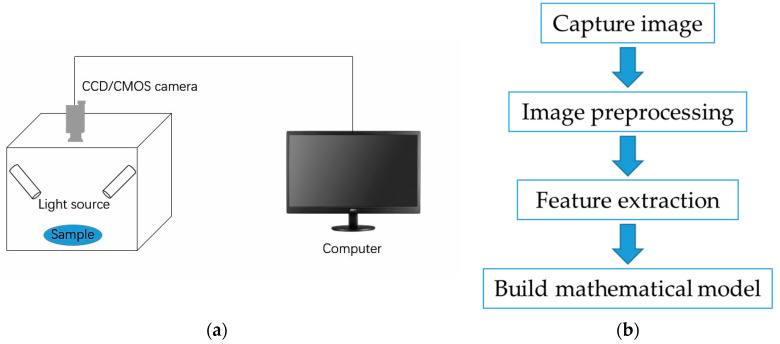
Machine vision detection system and its process: (**a**) machine vision detection system; (**b**) machine vision detection process.

**Figure 2:**
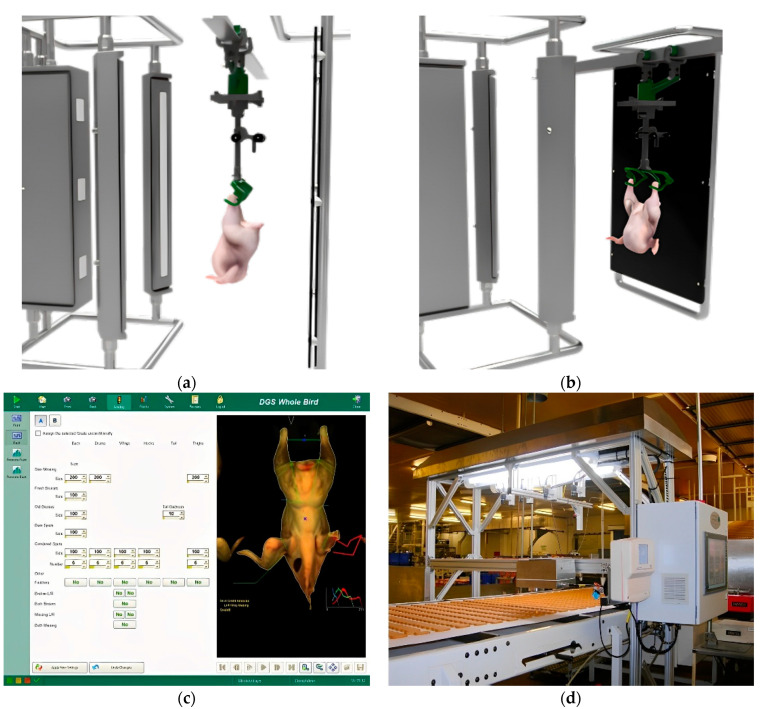
Non-destructive detection of machine vision system: (**a**,**b**) the hardware part of the machine vision inspection system for chickens; (**c**) the software part of the machine vision inspection system for chickens; (**d**) machine vision inspection system for cutting meat.

Machine vision technology involves capturing object images through cameras and converting the visual information into digital data through feature extraction, so as to obtain various features in the source image, and finally understand and make decisions on the detected object according to the discrimination criteria. In the context of livestock and poultry meat, machine vision technology has been proven to be effective in detecting characteristics such as marbling patterns [4], tenderness [5], color [6], and freshness [7]. The common process diagram of a machine vision detection system is illustrated in Figure 1. Non-destructive detection of machine vision systems is shown in Figure 2.

The characteristics of machine vision technology in meat quality detection can be summarized in the following three aspects:

1. Non-contact and non-destructive: Machine vision technology enables non-contact detection, ensuring that the object being measured remains uncontaminated and undamaged. It allows for real-time and long-term monitoring [8].
2. High sensitivity: This aspect is mainly reflected in the broader spectral range and finer resolution. While the human eye can perceive visible light in the wavelength range of 400–760 nm, machine vision can recognize visible light, ultraviolet (100–400 nm), infrared (760–0.3 mm), and more with the help of methods, expanding the detection capabilities across the spectrum. The captured images are composed of pixel points in an image matrix. Monochrome images have a minimum grayscale level of six bits per pixel, while color images have a minimum grayscale level of eight bits per pixel. The instrumentation-grade cameras easily provide 14–16-bit dynamic range. Digitizing at eight digits/pixel is typical for speed. An eight-bit image corresponds to pixel depths ranging from 0 to 255 levels, while the human eye can typically distinguish only about 40 levels. Generally speaking, cracks with a width of more than 0.1 mm can be recognized by the human eye, but machine vision technology can improve the recognition ability by more than 10–100 times [9].
3. Objectivity: The traditional manual detection methods rely on human expertise and visual detection, making the results susceptible to subjective factors and external environmental influences. In contrast, machine vision detection is not affected by detection conditions or operators, ensuring objective and efficient completion of the detection task using consistent evaluation criteria.

Machine vision detection offers advantages such as non-contact, high sensitivity, and objectivity, making it well suited for online quality detection of livestock and poultry meat. However, there are some challenges and areas for improvement that need to be addressed:

1. High environmental requirements: During the image acquisition stage, factors such as lighting conditions, environmental factors, shooting angles, and distances can affect the image features of the inspected objects, thereby impacting the detection accuracy. Additionally, image noise interference and partial occlusion of the inspected objects can also degrade the image quality, resulting in reduced detection accuracy. Finding ways to improve image acquisition quality and minimize the influence of external factors is an important challenge.
2. Large sample data: In practical image acquisition, a significant amount of sample data are often required, and the accuracy of the detection results is directly related to the volume of the sample data. Furthermore, constructing a database for meat quality assessment requires a diverse range of samples representing different quality levels. One of the future research directions is how to reduce the amount of sample data while ensuring the accuracy of quality detection.
3. Challenging feature extraction: For the accuracy and real-time performance of livestock and poultry meat quality detection, even if the detection algorithm is constantly updated, there is still a certain gap between the detection efficiency and accuracy of algorithms compared to the actual production requirements. Enhancing the fast and accurate extraction of image features to improve the accuracy and real-time capabilities of the detection system remains a current challenge.

### 2.2. Image Processing Technology

Machine vision technology generally involves processes such as image acquisition, processing, and analysis. Image processing encompasses analog image processing and digital image processing, emphasizing the use of various algorithms to transform and manipulate the color model of an image, representing a process from one image to another. Image analysis, on the other hand, focuses on segmenting, extracting, and measuring the objects of interest in an image to obtain their objective information, thereby describing the image in terms of data. While machine vision technology enables objective judgments of detected objects, compensating for the limitations of traditional human sensory perception, the quality of the obtained images significantly affects the results of machine vision. The image processing component plays a pivotal role throughout the entire machine vision process, and thus, the effectiveness of image processing directly impacts the final detection outcomes. In food image analysis, machine vision systems utilize color models to capture and analyze the visual characteristics of food products. They can effectively extract and analyze the relevant information from images by utilizing the conversion between different color models. In addition to color models and conversion algorithms, the utilization of image segmentation techniques to separate and extract region of interest (ROI) is also an indispensable process. By integrating suitable color models, conversion algorithms, and image segmentation techniques, machine vision systems can achieve accurate and efficient tasks based on visual features, such as quality control, grading, and classification.

#### 2.2.1. Color Model

**Figure 3:**
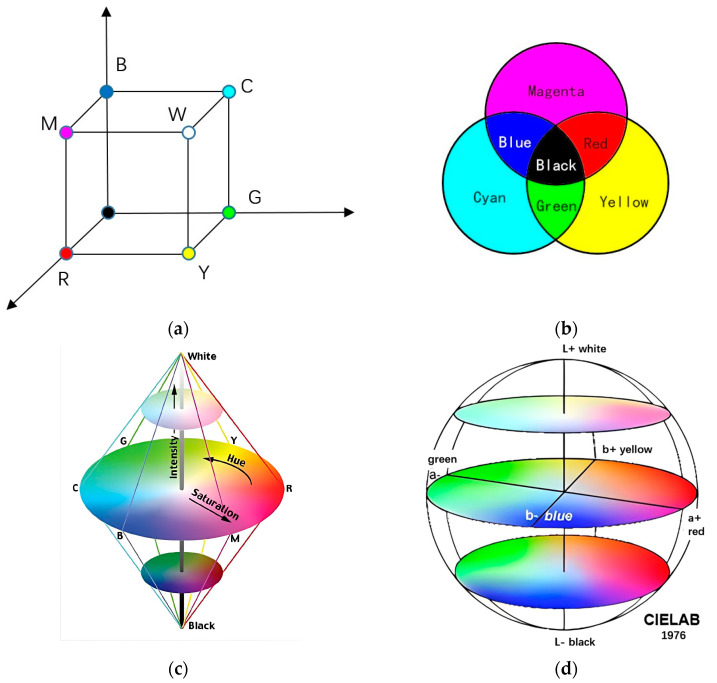
Four types of color models: (**a**) RGB color model; (**b**) CMY color model; (**c**) HIS color model; (**d**) CIE color model.

The color model refers to a subset of visible light within a three-dimensional color space, which encompasses all colors within a specific color gamut. In the field of food, there are four commonly used color models: RGB, CMY, HIS, and CIE. These color models are represented in Figure 3, illustrating their respective color spaces.

1. RGB color model: The RGB color model is a commonly used model for representing color information. Its color model equation is given by the following:
  (1) F=α(R)+β(G)+γ(B)

In the equation, α, β, and γ represent the mixing ratios of the red, green, and blue colors, respectively, known as the color coefficients [10]. This model quantitatively represents colors based on the brightness of the red, green, and blue components. The RGB color model follows the additive color principle of the primary colors, making it suitable for emissive devices such as displays [11]. For a given color, the RGB encoding values can be interpreted as coordinates in a three-dimensional space. These coordinates form a unit cube, as depicted in Figure 3a. The vertex points of the cube represent specific colors: (0, 0, 0) corresponds to black, (1, 1, 1) corresponds to white, and the other six vertices represent red (R), yellow (Y), green (G), cyan (C), blue (B), and magenta (M).

2. CMY color model: The CMY color model is a subtractive color model composed of cyan, magenta, and yellow as primary colors, as depicted in Figure 3b. Unlike the RGB color model, the CMY color model follows the subtractive color principle. The color model of CMY is almost identical to the RGB color model in terms of the corresponding subspaces.
3. HSI color model: When observing objects, the human eye is more sensitive to hue, saturation, and intensity. The HSI (hue, saturation, intensity) color space is designed to describe colors based on these three components [12], as depicted in Figure 3c. The establishment of the HSI model is based on two important principles: firstly, the intensity component (I) is independent of the color information in the image, and secondly, the hue (H) and saturation (S) components align better with the color characteristics perceived by the human eye [13]. This makes the HSI color model a useful tool for studying image processing algorithms, and thus it is commonly used in machine vision systems.
4. CIE color model: The CIE (International Commission on Illumination) color model is one of the earliest color models proposed by the commission, as depicted in Figure 3d. It is a three-dimensional model, where two dimensions define color and the third dimension defines brightness or luminance. The most commonly used CIE color models are CIE XYZ and CIE L*a*b*.

The CIE XYZ color model is derived from the theory of three primary colors (R, G, and B). It calculates the stimulus values of the three primary colors, X, Y, and Z in the CIE color space through the standard observer’s color matching functions $\bar{x\left(\lambda \right)},\;\bar{y\left(\lambda \right)}$, and $\bar{z\left(\lambda \right)}$. Any color can be represented as a combination of X, Y, and Z stimulus values, which are the coordinate values in the CIE color space representing the range of colors visible to humans [10].

The CIE L*a*b* color model is a device-independent color model that consists of a lightness parameter (L) and two color axes (a, b). It is designed to represent differences between colors and is considered a uniform color space [14]. The color space represented by CIE L*a*b* appears more visually uniform and closer to human perception compared to the RGB color space. The color feature of CIE L*a*b* is also the most commonly used feature in the significance detection and is more commonly used in the color detection of food [15].

#### 2.2.2. Conversion Algorithm between Color Models

The RGB color model is easy to understand and implement on hardware. However, since all three components are used to represent hue, changing the value of any one component will alter the overall color of the pixel. The CMY color model can mitigate color loss to some extent but operates at a slower speed, making it suitable for color printing. The HSI color model provides independent control over brightness and chromaticity, enabling better object segmentation even when external lighting conditions change. The CIE color model defines the largest color gamut, eliminating color loss issues. However, compared to other color models, it requires more pixel data to achieve the same color accuracy. Due to variations in color spaces among different devices capturing images and the need for efficient image processing and analysis, color model conversions are often necessary. Commonly used color model conversion algorithms include:

1. The algorithm for converting between the RGB and CMY color models is as follows:
  (2) C=255−R
  (3) M=255−G
  (4) Y=255−B
2. The algorithm for converting between the RGB and HSI color models is as follows:
  (5) $$I=\frac{1}{3}(R+G+B)$$
  (6) $$S=1-\frac{3}{R+G+B}[\min\left(R,\;G,\;B\right)]$$
  (7) $$H=arccos\left\{\frac{\frac{1}{2}[\left(R-G\right)+\left(R-B\right)]}{{{\left[(R-G\right)}^{2}+(R-B)(G-B)]}^{\frac{1}{2}}}\right\}$$
3. The algorithm for converting between the RGB and CIE XYZ color models is as follows:
  (8) $$\left(\begin{array}{c} X\\ Y\\ Z \end{array}\right)=\left(\begin{array}{ccc} 0.608 & 0.714 & 0.200\\ 0.299 & 0.587 & 0.144\\ 0.000 & 0.066 & 1.112 \end{array}\right)\left(\begin{array}{c} R\\ G\\ B \end{array}\right)$$
4. The algorithm for converting between the CIE XYZ and CIE L*a*b* color models is as follows:
  (9) $$L*=116f\left(Y/Y_{n}\right)-16$$
  (10) $$a*=500\left[f\left(X/X_{n}\right)-f\left(Y/Y_{n}\right)\right]$$
  (11) $$b*=200\left[f\left(Y/Y_{n}\right)-f\left(Z/Z_{n}\right)\right]$$

L is between 0 and 100, a and b are between −300 and +300. From −a to +a indicates the transition from green to red, and −b to +b indicates the transition from blue to yellow.

$X_{n},\;Y_{n},\;Z_{n}$ in the formula represent the white parameter values in X, Y, and Z, respectively.

(12) $$f\left(x\right)=\left\{\begin{array}{c} X^{1/3}\;\;\;\;\;\;\;\;\;\;\;\;\;\;\;x>0.008856\\ 7.787x+16/116\;\;\;\;x≦0.008856 \end{array}\right.$$

#### 2.2.3. Image Segmentation

Image segmentation is a part of image processing and serves as an initial step in image analysis. It involves classifying all the pixels in an image into several distinct regions with specific similarities, thereby dividing the image into mutually exclusive and connected regions [16]. Commonly used segmentation algorithms in image processing include threshold segmentation, edge-based segmentation, region-based segmentation, clustering analysis, wavelet transform-based segmentation, mathematical morphology-based segmentation, neural network-based segmentation, and genetic algorithm-based segmentation. Table 1 provides a comparison of the advantages, disadvantages, and application scopes of these eight segmentation methods.

### 2.3. Application of Machine Vision Technology on Quality Detection of Livestock and Poultry Meat

**Figure 4:**
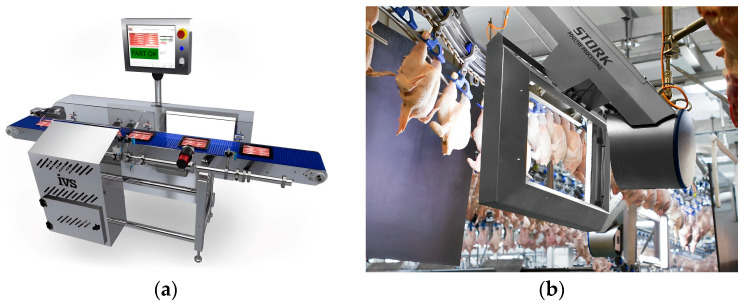
The application scenario of machine vision technology in livestock and poultry meat quality detection: (**a**) quality classification of pork; (**b**) quality detection of chicken carcass.

In recent years, machine vision and image processing techniques have gradually been applied to the quality detection of livestock and poultry meat. Key indicators for meat quality detection include freshness, meat color, intramuscular fat content (IMF%), volatile basic nitrogen, back fat thickness, drip loss, water-holding capacity, dry matter content, total fat content, protein content, and total bacterial count. Among these indicators, freshness, meat color, and IMF% have been successfully detected using machine vision methods by researchers. Figure 4 shows the application scenario of machine vision technology in livestock and poultry meat quality detection.

Freshness is considered one of the most crucial indicators for assessing meat quality and safety [17]. Luo et al. [18] employed machine vision technology and machine learning models to assess the freshness of beef. The study involved several steps. Initially, the ROI in the collected images was segmented, followed by preprocessing techniques such as filtering, denoising, and downsampling. Next, the Oriented Bounding Box algorithm and volume algorithm were used to determine the depth and volume of the processed images. Subsequently, a four-element viscoelastic model was established to fit the depth and volume data, capturing the mathematical characteristics of beef’s viscoelasticity. Using these mathematical features, a regression model was constructed and validated through experimental testing, resulting in the development of an optimal prediction model and method. The prediction model and method were utilized to determine the pH and total volatile basic nitrogen (TVB-N) content of beef. The results indicated that the calibration set and prediction set had correlation coefficients of 0.7636 and 0.9036 and 0.7669 and 0.8388, respectively. Xu et al. [19] proposed a novel olfactory visualization system for detecting the freshness of beef based on a colorimetric sensor array and chemometrics methods. Firstly, twelve color-sensitive materials were fixed on a hydrophobic platform to capture the odor information of beef samples based on the solvent-induced color change effect. Secondly, a machine vision algorithm was utilized to extract the odor fingerprints, and a principal component analysis was employed to compress the feature dimensions of the fingerprints. Finally, four qualitative models, namely k-nearest neighbors, extreme learning machine, support vector machine (SVM), and random forest, were constructed to evaluate the volatile basic nitrogen and total bacterial count, which characterized the freshness of beef. The results demonstrated that the SVM model exhibited favorable predictive capabilities, with accuracies of 95.83% for the training set and 95.00% for the prediction set. Jiang et al. [20] utilized machine vision technology for beef freshness detection. They studied the temporal changes in the R, G, and B color channel values of beef images. The research findings revealed that the R value decreased linearly with time, while the G and B values increased linearly with time. Based on the observed real-time changes, a beef freshness recognition model was developed. The accuracy of the model for detecting beef freshness was reported to be 90%. Zhang [21] proposed a method for grading the freshness of pork by integrating image features and olfactory features. Firstly, the ROI in the pork image was extracted, followed by preprocessing steps such as grayscale conversion, noise removal, and background segmentation. Subsequently, image features were extracted, which were further divided into color features and texture features. These two types of features were then fused together to obtain the fused color-texture feature. Simultaneously, the olfactory information of the same batch of pork was collected, and olfactory features were extracted. These olfactory features were then fused with the image features to obtain the image-olfactory fusion feature. Finally, different classification models, including the extreme learning machine, random forest, learning vector quantization neural network, and SVM model, were sequentially employed to achieve the freshness grading of pork based on color features, texture features, color–texture fusion features, olfactory features, and image-olfactory fusion features. The results showed that under the same feature type and different classification models, the classification model of SVM had the highest accuracy of 100%. In the case of the same classification model and different feature types, the classification model based on image olfactory fusion features had the highest accuracy of 100%.

Meat color is a visually influential factor that affects consumers’ purchasing decisions, and consumers often pay close attention to the color state of meat products [22]. Murashev et al. [23] have established an automated quality monitoring system for sausages. By analyzing the colors in the RGB images of sausages, they obtained the parameter values of individual color channels (R, G, or B) and their correlation with quality evaluation criteria such as pH, moisture content, and dry matter content. This system enables the monitoring of product quality based on these correlations. Lan et al. [24] utilized machine vision technology to extract feature parameters of beef marbling patterns. Subsequently, they further optimized these parameters using the local binary pattern algorithm. Finally, an intelligent grading model was established based on the convolutional neural network (CNN) algorithm. The results demonstrated that the grading accuracy achieved the following percentages: 84.2% for Grade 1, 89.4% for Grade 2, 81.9% for Grade 3, 84.1% for Grade 4, and 82.6% for Grade 5. Sun et al. [25] conducted research on using color features of pork images to detect pork color. Firstly, pork images were collected, and the background and muscle regions in the images were segmented. Then, color features were extracted from different color spaces, including RGB, HSI, and L*a*b*. Based on these extracted features, partial least squares regression (PLSR) and SVM models were established for analysis. Finally, different color detection models and their scores were obtained. The results showed that the highest detection accuracy for PLSR and SVM models was 90.9% and 93.3%, respectively. Sánchez et al. [26] proposed an approach for multivariate analysis of beef color changes using white-box machine learning techniques. Firstly, they used a computer vision system (CVS) to capture the color of the beef pieces. The differences between the three color spaces (RGB, HSV, and CIE Lab*) were then examined. Finally, the performance of three white-box classifiers (decision tree, logistic regression, and multiple normal distributions) for predicting color in both fresh and non-fresh beef was evaluated. The results showed that the best prediction was performed by using CIE Lab* and logistic regression, which used a plane to separate the fresh and non-fresh beef samples in the 3D color space (L*, a*, and b*).

The influence of IMF% is a crucial factor affecting consumers’ perceptions of food quality. Chen et al. [27] proposed an algorithm utilizing machine vision technology to estimate IMF%. A total of 1481 photographs of pigs’ loin muscles were subjected to computer vision scoring (IIMF%), followed by measurements of actual IMF%, meat color, marbling score, back fat thickness, pH value, and drip loss. Subsequently, stepwise regression (SR) and gradient boosting machines (GBMs) were employed to construct a predictive model for IMF%. The results revealed correlation coefficients of 0.68, 0.64, 0.48, 0.45, and 0.25 between IMF% and IIMF%, marbling score, back fat thickness, moisture content, and pH value, respectively. The predictive accuracy of the SR and GBM models, based on residual distribution, was determined to be 0.875 and 0.89, respectively. Munoz et al. [28] employed segmented images to investigate and compare the recognition accuracy of eight convolutional neural networks for muscle fat content. A total of 252 images were used for training, 61 for validation, and 62 for testing purposes. The results demonstrated that CNN3-512 achieved a pixel-wise accuracy of 0.99 and an accuracy close to 0.84 in determining IMF% using a limited number of training images. Barbon et al. [29] utilized the marbling pattern features in beef images to analyze the IMF%. Initially, the beef images were segmented using the Otsu method and underwent illumination normalization and contrast enhancement. The erosion technique was then applied to remove boundary pixels from the ROI to eliminate fat coverage. Subsequently, the marbling pattern features were extracted, and a detection model was established based on the k-Nearest Neighbor algorithm. The results indicated that the detection accuracy of the model for analyzing IMF% was 81.59%.

This section provides a detailed exposition of the principles, characteristics, limitations, and color models in image processing within the context of machine vision technology. It enumerates eight common image segmentation methods used in image processing and offers an overview of research progress in machine vision technology pertaining to the detection of freshness, meat color, and intramuscular fat content in livestock and poultry meat. These studies collectively illustrate the advancements achieved through machine vision technology in assessing the aforementioned meat attributes. The integration of sophisticated imaging techniques and machine learning algorithms has contributed to heightened precision and efficiency in evaluation. By scrutinizing subtle variations in color, texture, and fat distribution, the condition of meat products can be promptly assessed, facilitating timely decisions regarding processing and grading. Nevertheless, it is imperative to acknowledge that challenges persist, including accounting for the variability in equipment settings and meat characteristics. Ongoing research and development are essential for refining machine vision technology and enhancing its stability across diverse meat types, breeds, and environmental conditions.

## 3. Quality Detection of Livestock and Poultry Meat Based on Hyperspectral Technology

### 3.1. Hyperspectral Technology

**Figure 5:**
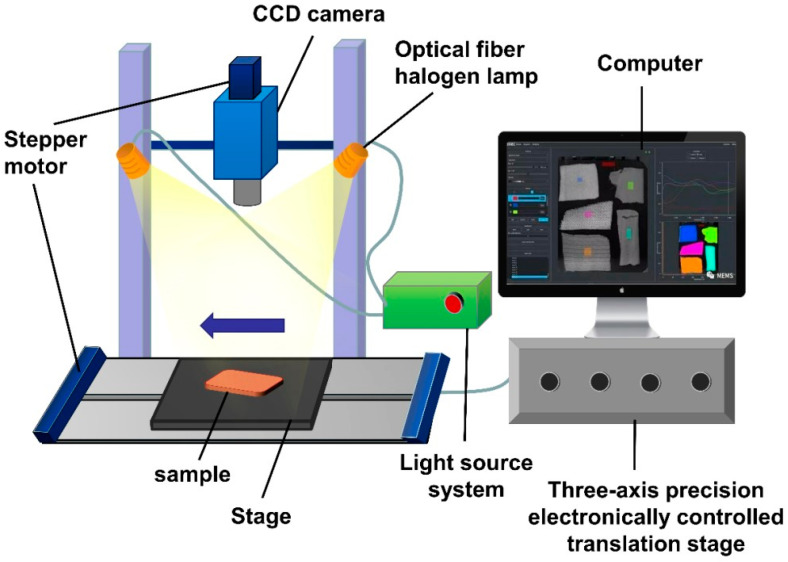
Common hyperspectral imaging systems.

**Figure 6:**
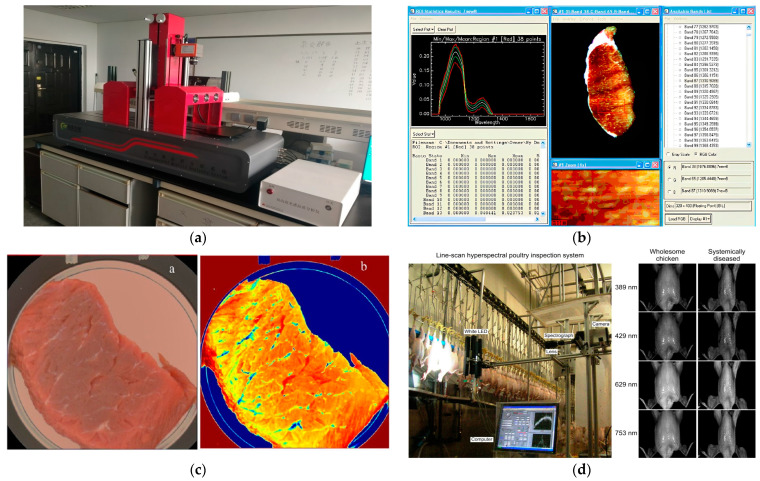
Non-destructive detection of hyperspectral system: (**a**) The equipment and instruments of hyperspectral systems; (**b**) The software operating interface of hyperspectral systems; (**c**) a. RGB image and b. hyperspectral image of pork; (**d**) line-scan hyperspectral reflectance imaging for online poultry carcass inspection.

Hyperspectral technology, as a type of spectral technology, combines the advantages of machine vision and multispectral imaging. It possesses the high-pixel imaging capability of a regular camera and the high-resolution imaging capability of a spectrometer. It can capture continuous images of samples at hundreds of wavelengths, simultaneously acquiring both the image information and the spectral information of the samples. Ultimately, a three-dimensional data cube is generated, consisting of two-dimensional images at different wavelengths [30]. Each pixel in this data cube contains spectral data at different wavelengths, representing the image information of the sample at each wavelength. The image information can reflect the visual texture features of the sample, while the spectral information can reveal its physical structure and chemical composition. Therefore, using hyperspectral technology for meat quality detection enables sensory evaluation based on surface physical features and provides insights into internal component content. The composition of a typical hyperspectral imaging system is illustrated in Figure 5 [31]. Non-destructive detection of hyperspectral systems is shown in Figure 6. Table 2 shows the comparative analysis between machine vision and hyperspectral in terms of resolution and spatial information, real-time processing, environmental adaptability, cost, and data volume.

### 3.2. Application of Hyperspectral Technology on Quality Detection of Livestock and Poultry Meat

**Figure 7:**
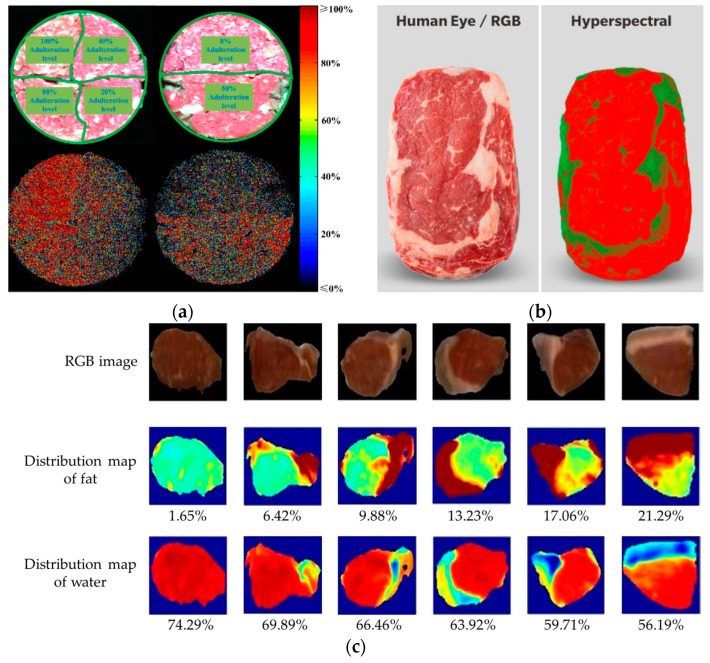
The application scenario of hyperspectral technology in livestock and poultry meat quality detection: (**a**) determination of meat adulteration; (**b**) determination of meat freshness; (**c**) determination of the physical characteristics and chemical composition of meat.

Hyperspectral technology integrates advanced disciplines such as optics, computer science, and electronics. It finds wide applications in various fields, including food safety detection [32], agricultural information remote sensing [33], drug component detection [34], and ecological environmental protection [35]. Research on non-destructive meat quality detection based on hyperspectral imaging technology mainly focuses on the determination of physical features and chemical composition of meat products, the rapid freshness assessment of meat, and adulteration detection in meat products [36,37,38,39]. Figure 7 shows the application scenario of hyperspectral technology in livestock and poultry meat quality detection.

Hyperspectral technology enables the rapid and non-destructive acquisition of physical and chemical indicators of samples, making it highly advantageous for industrial-scale online quality detection of livestock and poultry meat. Velasquez et al. [40] developed a system using hyperspectral technology to grade and classify the marbling pattern of beef based on the Japanese beef marbling standard. They digitally processed 12 grading standards to obtain shape and spatial distribution parameters for each marbling pattern grade. Subsequently, a hyperspectral imaging system operating in the 400–1000 nm range was used to scan 35 samples of the longest back muscle in reflectance mode. The 528 nm wavelength was selected for image segmentation of the samples and background, while the 440 nm wavelength was used for marbling pattern grading of the samples. A mathematical model was established to process and analyze the spectral information, and the experimental results showed an error rate of only 0.08% for this model. Kucha et al. [41] utilized hyperspectral imaging technology to detect IMF% in pork. The study involved gas chromatography (GC) analysis of the fatty acid profile and scanning of the minced pork section using a hyperspectral imaging system operating in the 900–1700 nm range. Texture features were extracted using mean spectral features, Gabor filter features, and wide line detector features. PLSR was employed to establish a correlation between these features and the GC results. A simplified model was developed using PLSR at selected wavelengths. The results indicated that the highest accuracy achieved by the detection model was 85.9%.

TVB-N is an important indicator for assessing the degree of spoilage in high-protein foods and is also used as a key parameter for evaluating the freshness of meat products [42]. In the process of spoilage in livestock and poultry meat, protein degradation leads to the formation of volatile amine compounds such as trimethylamine, dimethylamine, and ammonia. These substances are collectively referred to as TVB-N [43]. Baek et al. [44] utilized hyperspectral images of pork and its actual TVB-N content to construct a PLSR model. They combined this model with a feature selection method and designed a short-wave infrared hyperspectral imaging system. The spectral data were subjected to maximum normalization to obtain the optimal RF-PLSR model. Experimental results demonstrated that the optimal model exhibited correlation coefficients of 0.94 and 0.90 for calibration and prediction accuracy, respectively. This model can be used for the detection of TVB-N content in fresh pork. Zhuang et al. [45] employed fluorescence hyperspectral imaging technology to study the quality attributes of unfrozen pork. Based on the fluorescence spectra of TVB-N, pH, L*, a*, and b*, they established a PLSR model and compared it with a PLSR model based on visible/near-infrared hyperspectral imaging. The results showed that the detection model based on fluorescence spectra exhibited correlation coefficients of 0.9447, 0.9037, 0.6602, 0.8686, and 0.8699 for TVB-N, pH, L*, a*, and b*, respectively.

Adulteration and authentication are highly concerned with the quality detection of livestock and poultry meat [46]. To reduce the occurrence of meat adulteration, researchers have applied hyperspectral imaging technology to the detection and identification of adulteration in livestock and poultry meat. Zheng et al. [47] proposed a rapid and non-destructive method for detecting adulterated duck meat in lamb mince using a hyperspectral imaging system (400–1000 nm). Firstly, the spectral data of the samples were acquired, and the regions of interest were segmented and extracted. Then, a detection model was established based on the PLSR model. The SR, successive projections algorithm, and Savitzky–Golay (S-G) smoothing methods were used to select wavelengths, and the PLSR model was evaluated using these methods to determine the optimal wavelengths. The experimental results showed that the PLSR model with the optimal wavelength selection had a prediction determination coefficient of 0.98 and a prediction standard deviation of 2.51%. Xie et al. [48] utilized hyperspectral imaging technology to construct a visualized model for assessing the quality changes of braised beef during the frying process. The predictive correlation coefficients of moisture content and shear force for braised beef, as determined by this model, were 0.908 and 0.763, respectively. These findings indicate that hyperspectral imaging technology possesses the capability to detect complex components, such as seasoning, within meat products. Wang et al. [49] collected hyperspectral data from lamb meat samples originating from three different regions in China. The spectral data were preprocessed using the area normalization method, followed by feature wavelength selection. Subsequently, a classification model for differentiating the origin of lamb meat was established using PLSR. The results revealed that the accuracy of this model for detecting the origin of lamb meat was 90.48%. Miriam et al. [50] in order to distinguish the breed (Iberian or Iberian × Duroc) of acorn-fed pigs of Iberian ham, the spectra of the 60 samples (24 samples of 100% Iberian ham and 36 samples of Iberian × Duroc crossbreed ham) were recorded only for the fat, only for the muscle, or for the whole slice with two benchtop near-infrared (NIR) spectrometers and five portable spectrometers, including four portable NIR devices (VIAVI MicroNIR 1700 ES, TellSpec Enterprise Sensor, Thermo Fischer Scientific microPHAZIR, and Consumer hysics SCiO Sensor), and one RAMAN device. The results showed that, in general, the whole slice recording produced the best results for classification purposes. The SCiO device showed the highest percentages of correctly classified samples (97% in calibration and 92% in validation). The SCiO sensor also showed the highest percentages of success when the analyses were performed only on lean meat (97% in calibration and 83% in validation), while in the case of fat tissue. Raman technology showed the best discrimination capacity (96% and 78%). Therefore, near-infrared spectroscopy and Raman spectroscopy can be used to identify ham samples quickly, non-invasively, and inexpensively according to variety purity.

This section elucidates the fundamental principles of hyperspectral technology, analyzes its merits in meat inspection, and provides an overview of research advancements in hyperspectral technology concerning the analysis of physical characteristics, chemical composition, meat freshness, and adulteration in livestock and poultry meat. These studies underscore the potential of hyperspectral technology to capture intricate details pertaining to meat attributes, encompassing physicochemical indicators like moisture content, protein distribution, and the identification of specific adulterants. The non-destructive nature of hyperspectral analysis facilitates a comprehensive evaluation of meat products without compromising their integrity. However, akin to pioneering technologies, challenges persist, such as the classification of detection models and the complexity of predictive variables. Hyperspectral technology demands robust calibration models to effectively correlate spectral data with meat properties across diverse samples and conditions. Moreover, the key to seamless application of hyperspectral technology in non-destructive meat quality assessment lies in refining techniques to eliminate extraneous data and accurately selecting feature wavelengths to enhance detection precision and computational efficiency. Despite these challenges, the advancements in hyperspectral technology present a transformative opportunity for the meat industry, with the potential to play a pivotal role in ensuring the authenticity, freshness, and safety of meat products.

## 4. Quality Detection of Livestock and Poultry Meat Based on Multi-Source Information Fusion Technology

### 4.1. Multi-Source Information Fusion Technology

**Figure 8:**
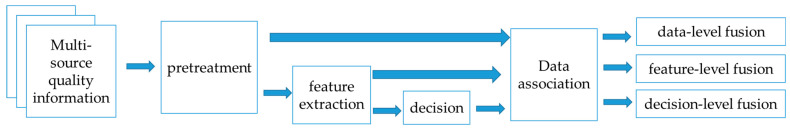
Diagram illustrating the process of information fusion in three levels.

**Figure 9:**
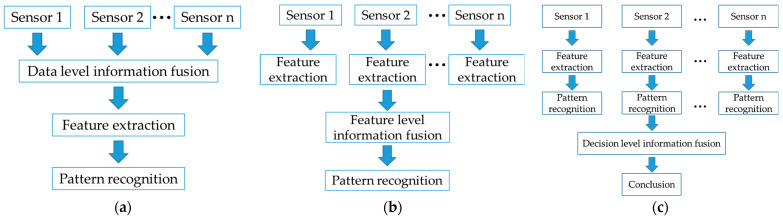
Process of fusion processing: (**a**) process of data-level fusion processing; (**b**) process of feature-level fusion processing; (**c**) process of decision-level fusion processing.

Multi-source information fusion technology refers to the rational selection, extraction, optimization, and combination of different types of information collected from various sensors, aiming to make more accurate decisions. In the process of quality detection of livestock and poultry meat, using a single type of sensor to acquire quality information can lead to certain limitations and biases. Single-modality data alone cannot accurately, comprehensively, and objectively reflect the true quality of livestock and poultry meat [51]. On the other hand, multiple heterogeneous sensors can evaluate the same sample in different feature spaces, providing more abundant, comprehensive, and complete information compared to multiple homogeneous sensors. Based on the levels of data abstraction in multi-sensor information fusion methods, the fusion of multi-source quality information can be divided into data-level fusion, feature-level fusion, and decision-level fusion [3]. The fusion processes of these three levels of information are illustrated in Figure 8, while the fusion processing for each level is shown in Figure 9. Multi-source information fusion methods can be broadly classified into classical information fusion and modern information fusion. Classical information fusion methods are based on theories such as Bayesian inference, weighted averaging, and Dempster-Shafer evidence theory. On the other hand, modern information fusion methods, mainly represented by neural networks and artificial intelligence, draw inspiration from the way the human brain processes information. These methods have seen rapid development and are gradually being applied to the quality detection of livestock and poultry meat [42].

### 4.2. Application of Multi-Source Information Fusion Technology on Quality Detection of Livestock and Poultry Meat

In recent years, with the continuous development of sensor technology, multi-source information fusion techniques based on multiple sensor information have also advanced and gradually found applications in detecting the freshness, physical properties, chemical composition, and adulteration of livestock and poultry meat. Compared to using a single machine vision or hyperspectral detection technique, multi-source information fusion technology exhibits more accurate detection results, stronger adaptability to material conditions, and reduced susceptibility to external environmental influences.

**Figure 10:**
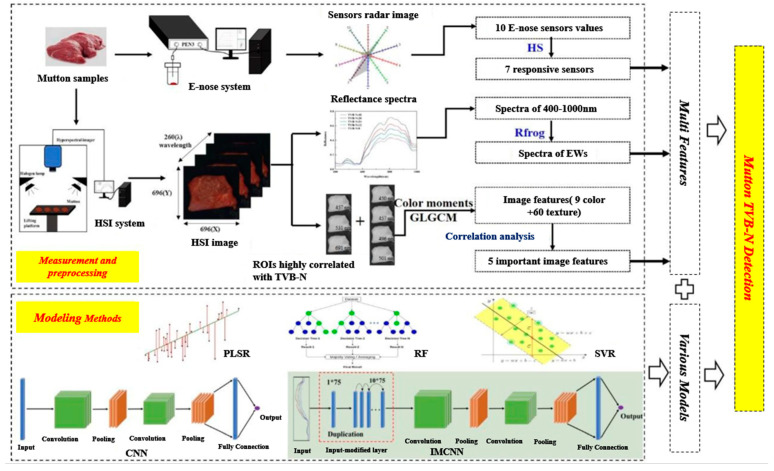
Flowchart of detection of mutton TVB-N using E-nose and HSI [52]. Reproduced or adapted from [52], with permission from *Food chemistry*, 2022.

In the field of detecting the freshness of livestock and poultry meat, scholars have already utilized multi-source information fusion techniques. Liu et al. [52] proposed a precise analysis method for assessing the freshness of lamb (Figure 10). Firstly, data from an electronic nose (E-nose) sensor was collected, followed by the extraction of reflectance spectra and image features from lamb hyperspectral imaging. Relevant variables were selected from these features. Subsequently, the electronic nose data, hyperspectral imaging, and CNN algorithm were integrated to establish a lamb freshness prediction model. The results indicated that the root mean square error of the prediction set for TVB-N content was 3.039 mg/100 g, with a correlation coefficient of 0.920 and a performance deviation ratio of 3.59. Weng et al. [53] proposed a method for capturing and integrating freshness parameters of livestock and poultry meat by combining E-nose, computer vision (CV), and artificial touch (AT) sensory technologies. Initially, odor features, color features, and elasticity features of the samples were extracted using E-nose, CV, and AT, respectively. Subsequently, a normalization algorithm was applied to unify the dimensionality of the three types of feature data, followed by feature-level fusion. A PLSR prediction model for TVB-N content was then established based on the fused data. Experimental results demonstrated that the model achieved prediction accuracies of 0.91 and 0.94 for pork and chicken meat, respectively. Zhu et al. [54] conducted a study on lamb freshness using hyperspectral imaging and near-infrared spectroscopy imaging. They collected freshness data for three different grades of lamb meat according to comprehensive evaluation standards for meat quality. The data was preprocessed using the S-G smoothing method combined with the first derivative. They applied competitive adaptive reweighting sampling, genetic algorithms, and successive projection algorithms to select feature variables from the full wavelength range. Subsequently, they established freshness detection models based on full-band variables, feature variables, and fused variables for hyperspectral imaging, near-infrared spectroscopy imaging, and fused imaging, respectively. A comparative analysis was conducted on the nine detection models established. The results showed that the fusion imaging model based on fused variables exhibited the best detection performance, with a detection accuracy of 100%.

**Figure 11:**
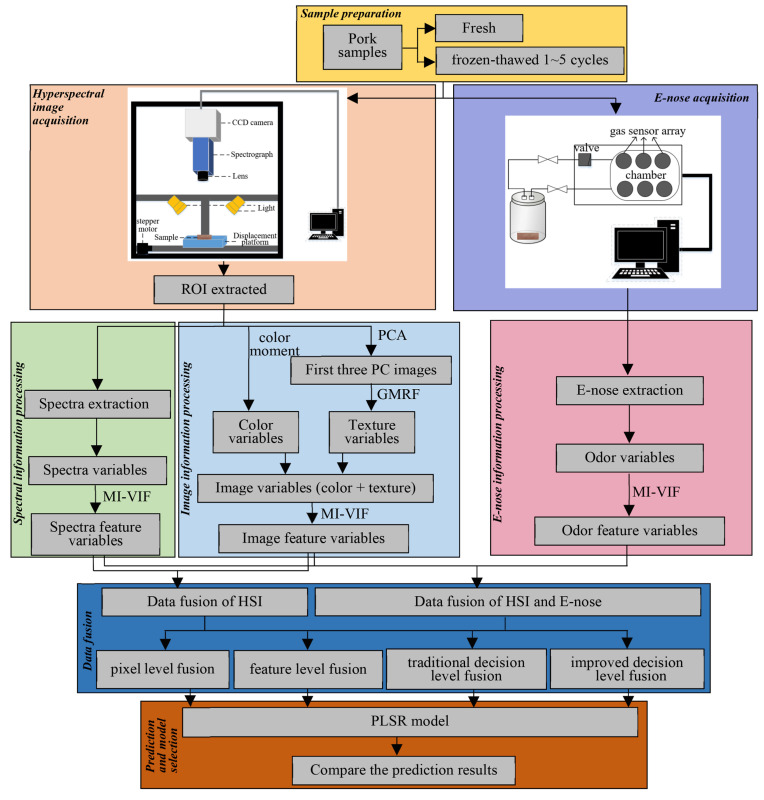
A data fusion method combining hyperspectral imaging and E-nose technologies for detecting moisture content (MC) in frozen pork [55]. Reproduced or adapted from [55], with permission from *LWT, Food Science and Technology*, 2022.

Multi-source quality information fusion technology has also shown excellent performance in detecting the physical properties and chemical composition of livestock and poultry meat. Cheng et al. [55] proposed a data fusion method combining hyperspectral imaging and E-nose technologies for detecting moisture content (MC) in frozen pork (Figure 11). They first collected data from 240 pork samples using hyperspectral imaging and E-nose technologies. Then, spectral and image information was extracted from the hyperspectral imaging data, while odor information was extracted from the E-nose data. Finally, the three types of information were fused through decision fusion to establish a fusion detection model. The detection results showed that the model achieved a correlation coefficient of 0.9533 and a root mean square error of 0.3869 on the prediction set. This method improved the prediction performance of MC in frozen pork. Aheto et al. [56] fused the texture and spectral information from hyperspectral imaging to predict the levels of 2-thiobarbituric acid reactive substances (TBARS) and peroxide value (PV) in pork muscle under different processing conditions. They first established PLSR models separately for the full spectrum and optimal spectrum. Then, they fused the spectral image and optimal spectral information to build a data fusion model. The results showed that for TBARS measurement, the model based on the optimal spectrum had a prediction determination coefficient of 0.896 and a root mean square error of prediction (RMSEP) of 1.034, outperforming the data fusion model. For PV measurement, the data fusion model yielded the best results, with a prediction determination coefficient of 0.899 and an RMSEP of 0.966. To further improve the prediction accuracy, Aheto et al. [57] integrated electronic nose technology to detect IMF% and PV in pork products. They first collected spectral data, image data, and electronic nose data from the samples. Based on the SVM model, they established models for median spectral features (MSF) from hyperspectral data, image texture features (ITF) from images, and average signal values (MSV) from electronic nose data. They then selected the optimal wavelengths highly correlated with IMF% and PV from MSF and ITF, respectively, and combined them to form “combined attribute features” (CAF). Finally, they fused CAF with MSV to achieve better prediction accuracy. The experimental results showed that the fused model achieved the highest prediction accuracy, with calibration and prediction correlation coefficients of 0.936 and 0.955 for IMF% and 0.895 and 0.901 for PV, respectively.

**Figure 12:**
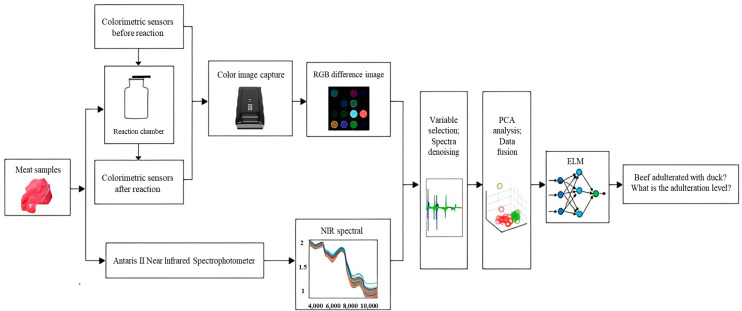
The sketch diagram of combing E-nose and FT-NIR to detect the beef adulterated with duck.

Multi-source quality information fusion technology has also shown great potential for detecting adulteration in meat products. T. Wang [58] utilized ultrasound technology and hyperspectral imaging to detect adulteration in processed beef steaks. They first collected ultrasound images and hyperspectral images of both authentic and synthetic beef steaks. Texture features were extracted from the images using the gray-level co-occurrence matrix method. They then established a data fusion identification model based on the texture features of both types of images. The identification model was further optimized, and the optimized model achieved a recognition rate of 100.00% for both the calibration and prediction sets. Pu et al. [59] employed hyperspectral imaging technology combined with CNN to differentiate between fresh and frozen-thawed beef samples. They utilized CNN to extract spectral and texture features and established a feature fusion model. The results showed that the model achieved a recognition accuracy of 88.89%. Han et al. [60] proposed a rapid method for detecting adulteration of duck meat in beef (Figure 12). They first analyzed the differences in basic characteristics and volatile organic compounds between raw beef and duck meat. Then, they established E-nose detection models for these characteristics and Fourier transform near-infrared (FT-NIR) spectroscopy detection models, as well as a data fusion detection model combining FT-NIR and E-nose. The results showed that the accuracy of FT-NIR reached 100% in independent sample detection of raw beef, beef-duck meat mixture, and raw duck meat. In the detection of adulteration, the correlation coefficient for FT-NIR detection was 0.913, and for E-nose detection, it was 0.841. However, when using the data fusion detection of E-nose and FT-NIR, the correlation coefficient increased to 0.972.

This section provides a comprehensive exposition of the principles, characteristics, fusion modes, and fusion methods of multi-source quality information fusion technology. It presents an overview of the research progress in the application of multi-source quality information fusion technology to the assessment of freshness, physical properties, chemical composition, and detection of adulteration in livestock and poultry meat. An increasing number of practical applications underscore the efficacy of multi-source quality information fusion technology in compensating for the limitations of singular detection methods by integrating data from diverse sources. This integration enhances detection precision, improves adaptability to material states, and augments resistance to external interferences, thereby offering a comprehensive approach to meat quality assessment that enables a more accurate and nuanced understanding of product attributes. Information fusion not only elevates measurement accuracy but also mitigates the inherent constraints of individual techniques. The utilization of advanced data fusion algorithms, including those based on machine learning and artificial intelligence, aids in extracting relevant data from complex datasets, supporting real-time decision-making along the entire meat supply chain. Nonetheless, challenges exist in coordinating different data sources and ensuring the reliability and consistency of fusion outcomes, presenting certain complexities. As research in this domain advances, collaboration between experts in fields such as food science and data analysis will be pivotal in optimizing the fusion process and establishing dependable protocols for quality assessment. The continuous refinement and integration of multi-source quality information fusion technology hold the potential to definitively ensure meat quality, enhance consumer confidence, and drive advancements in the realm of food safety.

## 5. Future Research Directions

The rapid operation of modern science and technology has increased the diversity of detection means; the advantages of various non-destructive detection technologies have been fully exploited; and the research on food safety detection, such as livestock and poultry meat, has also been more in-depth. Based on machine vision, hyperspectral imaging, and multi-source quality information fusion technology, the detection method for livestock and poultry meat has demonstrated certain advantages over traditional methods. It has also achieved significant progress, indicating its enormous potential in the field of livestock and poultry meat quality detection. However, upon summarizing the aforementioned detection technologies, certain issues have been identified. Thus, it is necessary to address these problems and provide prospects for future improvements.

First of all, due to the interdisciplinary nature of machine vision technology and the complexity of detection systems, the related algorithms are not yet fully mature, so when machine vision conducts high-precision detection such as defect detection in meat, these defects may be very small and difficult to detect on different meat surfaces, increasing the difficulty of detection. Additionally, the acquisition and processing of image information are susceptible to external factors such as light sources, conveyor systems, the number of cameras, and the color and texture of different meats. These factors can result in certain deviations in the detection results, limiting the analysis to the surface characteristics of the samples while being unable to assess internal chemical quality. Therefore, the future focus of research in machine vision technology should be on simplifying and optimizing algorithms, reducing the impact of external environmental factors. This includes selecting and extracting the relevant features of interest to build suitable models and eliminating redundant weights and connections to reduce the size and computational complexity of the model. Improving the cleanliness of the detection area, developing specific lighting techniques for different types of samples, and placing the camera in a vibration-free or low-vibration environment can enhance defect visibility or eliminate image noise, thereby improving the efficiency and stability of machine vision systems.

In addition, hyperspectral technology provides high data accuracy, unified spectra, and superior spatial distribution capabilities. However, the equipment used for hyperspectral imaging is often large and expensive, and it has poor resistance to interference, which can add to the cost burden in the meat industry. At the same time, the spectral characteristics of meat are affected by many factors, including moisture, fat content, etc. The processing of hyperspectral data are relatively complicated, and a large amount of data generated by hyperspectral imaging needs dimensionality reduction, which reduces the processing speed. Therefore, future research directions for the development of hyperspectral technology should focus on enhancing anti-interference capabilities and improving computational efficiency. For example, conducting professional site planning and optimizing sensor design before data acquisition to minimize external interference, employing stable and efficient algorithms for noise reduction, selecting the most effective feature wavelengths, and establishing high-precision discriminative models all contribute to improving computational efficiency.

Finally, the future development trend of livestock and poultry meat quality detection lies in online real-time monitoring, enabling the acquisition of information from the entire surface and real-time processing of image data. Although multi-source quality information fusion technology has shown improved results, the processing after fusion requires a substantial database, and the computational load of the fused model increases significantly. Therefore, the future focus of research is to establish a rapid, stable, and efficient quality detection system for poultry and livestock meat based on multi-source quality information fusion technology. For example, when selecting sensors, sensitivity, resolution, and adaptability should be considered to ensure comprehensive and accurate capture of meat quality features. Consider the application of deep learning techniques, such as CNN or Recurrent Neural Network (RNN), to learn features from large datasets and enhance the system’s capability to identify complex quality features. Also, consider establishing a cloud-based platform for centralized data management and analysis to improve the overall efficiency of the system.

## Figures and Tables

**Table 1:** Advantages, disadvantages, and application scopes of eight image segmentation methods.

Method	Advantage	Disadvantage	Application Scope
Threshold segmentation	The calculation process is simple, with high computational efficiency and fast speed.	It is sensitive to noise and has relatively low robustness.	It is suitable for images where there is a significant contrast between the target and background.
Edge-based segmentation	The edge localization is accurate, and the process is fast in terms of speed.	It cannot guarantee the continuity and closedness of the edges.	It is suitable for images with low noise interference and significant edge variations.
Region-based segmentation	The image segmentation has a large spatial scope and exhibits distinct regional features.	It may lead to over-segmentation of the image.	It is more suitable for images that possess a well-defined regional structure.
Clustering analysis-based segmentation	The approach is characterized by its simplicity, ease of implementation, fast convergence speed, and ability to reach local optima efficiently.	It is insensitive to noise and uneven grayscale.	It is suitable for images that exhibit uncertainty and ambiguity.
Wavelet transform-based segmentation	The method can effectively extract information from signals and is not sensitive to noise.	In the face of different real-world situations, it is necessary to choose appropriate filtering functions to effectively perform image segmentation.	It is used for edge detection and can extract multi-scale edges. Additionally, it can differentiate edge types by calculating and estimating the image’s singularity.
Mathematical morphology-based segmentation	It achieves good localization results, high segmentation accuracy, and exhibits good noise resistance.	After image processing, there may still exist numerous short lines and isolated points that do not correspond to the target.	It is suitable for tasks such as noise suppression, feature extraction, and edge detection in image processing.
Neural network-based segmentation	It can effectively address noise and unevenness issues in images.	It requires a large amount of data, operates at a relatively slow speed, and has a complex structure.	It is suitable for handling problems such as noise suppression and unevenness in images.
Genetic algorithm-based segmentation	Genetic algorithms possess strong global optimization search abilities.	The selection of different fitness functions, as well as the determination of crossover and mutation probabilities, can impact the segmentation results.	It is suitable for threshold-based segmentation methods and region-growing methods, aiming to find the global optimum in segmentation.

**Table 2:** The comparative analysis between machine vision and hyperspectral in terms of resolution and spatial information, real-time processing, environmental adaptability, cost, and data volume.

Method	Resolution and Spatial Information	Real-Time Processing	Environmental Adaptability	Cost	Data Volume
Machine vision	High spatial resolution	Excellent real-time processing capability.	It is well adapted to various lighting and environmental conditions.	Low cost	Small data volumes
Hyperspectral	Low spatial resolution	Poor real-time processing capability.	More sensitive, requiring complex calibration under changing conditions.	High cost	Large data volumes

## Data Availability

The data presented in this study are available on request from the corresponding author. The data are not publicly available due to commercial reason.
